# Impact on Bacterial Resistance of Therapeutically Nonequivalent Generics: The Case of Piperacillin-Tazobactam

**DOI:** 10.1371/journal.pone.0155806

**Published:** 2016-05-18

**Authors:** Carlos A. Rodriguez, Maria Agudelo, Yudy A. Aguilar, Andres F. Zuluaga, Omar Vesga

**Affiliations:** 1 GRIPE (*Grupo Investigador de Problemas en Enfermedades Infecciosas*), Facultad de Medicina, Universidad de Antioquia, Medellín, Colombia; 2 Infectious Diseases Unit, Hospital Universitario San Vicente Fundación, Medellín, Colombia; Institute for Bioengineering of Catalonia, SPAIN

## Abstract

Previous studies have demonstrated that pharmaceutical equivalence and pharmacokinetic equivalence of generic antibiotics are necessary but not sufficient conditions to guarantee therapeutic equivalence (better called pharmacodynamic equivalence). In addition, there is scientific evidence suggesting a direct link between pharmacodynamic nonequivalence of generic vancomycin and promotion of resistance in *Staphylococcus aureus*. To find out if even subtle deviations from the expected pharmacodynamic behavior with respect to the innovator could favor resistance, we studied a generic product of piperacillin-tazobactam characterized by pharmaceutical and pharmacokinetic equivalence but a faulty fit of Hill’s *E*_*max*_ sigmoid model that could be interpreted as pharmacodynamic nonequivalence. We determined the impact *in vivo* of this generic product on the resistance of a mixed *Escherichia coli* population composed of ∼99% susceptible cells (ATCC 35218 strain) and a ∼1% isogenic resistant subpopulation that overproduces TEM-1 β-lactamase. After only 24 hours of treatment in the neutropenic murine thigh infection model, the generic amplified the resistant subpopulation up to 20-times compared with the innovator, following an inverted-U dose-response relationship. These findings highlight the critical role of therapeutic nonequivalence of generic antibiotics as a key factor contributing to the global problem of bacterial resistance.

## Introduction

The rise of antimicrobial resistance is a public health emergency that is threatening the conquests of modern medicine with potentially dire consequences for humankind if not addressed promptly [1–4]. The widespread use and misuse of antibiotics has exerted an enormous selective pressure on microorganisms, leading to the emergence of resistance to every single known antibacterial drug [5], especially in Gram negative bacilli, for which very few antibiotics have been approved in the last decades [6].

Besides factors like prescription without indication [7], unjustified prolonged therapies, inappropriate dosing, disregard of the pharmacodynamics, poor adherence, and abuse of antibiotics by the agriculture and animal industry [8], there is a key factor that has not been considered: the use of generic products that fail therapeutic equivalence. Nevertheless, this point conveys the greatest relevance given that the vast majority of drugs consumed worldwide is produced by generic makers, for instance, close to 100% in China, India and Brazil; 70%-90% in USA, Germany, Canada and UK, and 30% in Japan [9].

We argue that generic antimicrobials are key determinants of resistance because our previous studies demonstrate that pharmaceutical equivalence, the only requirement of regulatory agencies to approve generic intravenous antibiotics [10], is a necessary but not sufficient condition for therapeutic equivalence, and most generic products of antimicrobials as important as vancomycin, oxacillin, gentamicin and meropenem failed therapeutic equivalence in validated animal models of human infection [11–14].

Only two groups have tried to reproduce our findings generic antibiotics, and both with published negative results. However, there are important methodological differences and limitations that explain the outcomes. The first paper was published in 2013 by Tattevin et al. [15] using the rabbit *Staphylococcus aureus* endocarditis model with six vancomycin generics manufactured in the U.S.A. and Europe, and found no differences between products. However, their model and analysis had several limitations: first, the CFU/g of vegetation in the untreated controls ranged from 7 to 10 log_10_, a 1000-fold range, with a SD of 0.8 log_10_ (in contrast with the thigh model where the usual SD in controls is <0.1 log_10_). The variation in the treated groups was also huge, ranging from 2 to 8 log_10_ after 5 days of therapy with a SD of ∼2 log_10_. With this variation, the power of the design to detect a difference of 1, 2, and 3 log_10_ in efficacy between products using 10 animals per group is 11%, 32%, and 69%, respectively (SigmaPlot 12.3, Systat Software Inc). Thus, a heavily underpowered model in addition to the use of parametric statistic tests with non-Gaussian data, explain the failure of the experimental design to find significant differences [16]. The second paper is a study by Louie et al. published in January 2015 [17], reporting the results from the evaluation of 6 vancomycin generics with FDA-demonstrated pharmaceutical equivalence: Hospira, Pfizer, APP, Sandoz, Baxter, and Mylan (Bioniche) in the mouse thigh infection model, trying to follow the methods employed by our group in the 2010 vancomycin paper [11]. The authors did not find differences across the products with regard to any *in vitro* evaluation or pharmacokinetic parameters, and the *in vivo* model yielded similar efficacy and potency. Although Louie et al. aimed to replicate our methodology they failed to do so. First, we used nonlinear regression and global curve-fitting analysis with thorough regression diagnostic criteria (adjR^2^, standard error of estimate, significance of parameters, normality, homoscedasticity, and absence of multicollinearity), whereas Louie’s group only reported the R^2^ and the estimate of maximum effect (E_max_) and effective concentration 50 (EC_50_) with their confidence intervals, showing parameters (in several generics) lacking statistical significance. Second, Louie et al. injected vancomycin q6h while we used a q1h dosing schedule. Considering that vancomycin is a time-dependent antibiotic with persistent effects (PAE) against *S*. *aureus* of 0.2 to 2 hours and that its elimination half-life in mice is ∼30 minutes, a q6h dosing interval is too long to adequately assess the pharmacodynamics because it puts all products in disadvantage.

These differences in analytic tools and experimental design preclude a direct comparison of our results to theirs, and thus, more research in the field is required and encouraged.

We have also shown that therapeutically nonequivalent generics of vancomycin enriched resistant subpopulations of *Staphylococcus aureus in vivo*, while the innovator actually reduced them [18]. However, *S*. *aureus* and vancomycin are not an ideal pair to study the influence of therapeutic nonequivalence on bacterial resistance because vancomycin resistant cells are quite uncommon and emerge very slowly, and the resistance mechanism (cell wall thickening) is complex and only partially elucidated [19, 20]. A more suitable model to test further the hypothesis that therapeutic nonequivalence promotes resistance will include a drug-microorganism pair similar to vancomycin and *S*. *aureus* in terms of clinical significance, but with a prompt and well-defined mechanism of resistance.

First, we deemed necessary to demonstrate, as expected, that a generic antibiotic with established pharmaceutical, pharmacokinetic (PK) and pharmacodynamic (PD) equivalence must select resistant bacteria in the same proportion and by identical mechanisms as the innovator, and it was in fact proved with a generic product of ciprofloxacin against *Pseudomonas aeruginosa* during seven days of exposure in the hollow-fiber pharmacodynamic system [21]. To test the opposite, i.e., if a generic antibiotic without therapeutic equivalence would favor resistance in a higher degree than the innovator, we studied *in vivo* the therapeutic equivalence of four generic products of piperacillin-tazobactam (TZP) against *Escherichia coli* ATCC 35218, a strain producing the plasmidic class A TEM-1 β-lactamase. In a second step, we assessed *in vivo* the impact of TZP on a mixed bacterial population containing a majority of susceptible individuals and a small fraction of resistant cells overexpressing the β-lactamase, comparing the only nonequivalent generic with the innovator.

The verification of our hypothesis that therapeutic nonequivalence promotes resistance would entail important consequences: first, it would indicate that the use of “bioequivalent” generics that fail therapeutic equivalence may be one of the factors contributing worldwide to the problem of antimicrobial resistance, emphasizing the need to revise current regulations for generic approval to include demonstration of *in vivo* efficacy; and second, if therapeutic equivalence ensures the same resistance selection profile of the innovator, the animal model would be a thorough proof for any generic antimicrobial that, by identifying therapeutically nonequivalent products, would prevent therapeutic failures and resistance. These benefits will certainly reduce the cost of bringing generics to clinical use.

## Materials and Methods

### Bacterial strains

For therapeutic equivalence experiments, we used *E*. *coli* ATCC 35218, a strain that produces a plasmid-encoded TEM-1 β-lactamase; this is the parental microorganism. A less-susceptible isogenic derivative, *E*. *coli* 35218R was obtained after serial passage of a high inoculum (8 log_10_ CFU/mL) of the parental strain on agar with 10/1.25 mg/L of the innovator product of piperacillin/tazobactam. A plasmid-cured derivative, *E*. *coli* 35218Δ*bla*, was obtained after incubation with sodium dodecyl sulfate (SDS) for 48 hours [22] and identified by replica-plating on plates with and without 64 mg/L of ampicillin. The absence of the *bla*_*TEM-1*_ gene was confirmed by PCR (see below).

### Media

Trypticase soy broth (TSB) or brain-heart infusion (BHI), and Mueller-Hinton agar (MHA) were the general culture media (Difco, Becton-Dickinson, USA). Cation-adjusted Mueller-Hinton broth (CA-MHB) was employed for *in vitro* susceptibility testing. In resistance selection experiments, MHA with 10/1.25 mg/L of piperacillin/tazobactam (innovator product) was used to quantify the resistant subpopulation. These concentrations of piperacillin and tazobactam correspond to 2.5-times the MIC of the parental strain using a fixed 8:1 piperacillin:tazobactam ratio.

### TZP products and *in vitro* activity

The study included the innovator of TZP (Wyeth, Catania, Italy; lots AIDL/11, AHFV/21 and AHJI/11) and four generics marketed by Farmalogica (Bogota, Colombia; lot 1251112), Procaps (Barranquilla, Colombia; lot 2061111), Farmionni (Barranquilla, Colombia, lot 2010642) and Vitalis (Bogota, Colombia, lot 0120049); all products were licensed for human use by the Colombian drug regulatory agency (INVIMA). The pharmaceutical, pharmacokinetic, and therapeutic equivalences of the generic TZP from Procaps was demonstrated in a previous study [23], the generics from Farmionni and Vitalis were only tested *in vivo*. For *in vitro* activity, the MIC of Wyeth and Farmalogica TZP (in a fixed 8:1 ratio) was determined by duplicate broth microdilution against *E*. *coli* ATCC 35218, *E*. *coli* 35218R and *E*. *coli* 35218Δ*bla* using the standard double dilution design. Additionally, the MIC was determined following the arithmetic dilution used by Jones et al. [24]. Unless specified otherwise, the doses and concentrations refer to the piperacillin component of TZP.

### Characterization of the strain *E*. *coli* 35218R

#### Susceptibility to other β-lactams

To better characterize the resistance profile of the strain 35218R, we performed automatic susceptibility testing (Vitek®, bioMérieux, France) to cefoxitin, ceftriaxone, ceftazidime, cefepime, aztreonam, ertapenem, imipenem, and meropenem.

#### Population analysis profile

The total population and the subpopulations of *E*. *coli* ATCC 35218 and 35218R able to grow on MHA with 1, 2, 4, 8, 16, 32, 64, 128, 256 and 512 mg/L of piperacillin (with tazobactam in a fixed 8:1 ratio) were assessed, comparing the innovator TZP (Wyeth) with the generic that failed therapeutic equivalence (TZP Farmalogica). A log-phase broth culture containing ∼8 log_10_ CFU/mL was plated on MHA with or without antibiotic and incubated for 48 hours at 37°C. The resistance frequency at each concentration was determined by dividing the number of resistant cells by the total population after three independent assays. Hill’s equation was fitted to the concentration-resistance frequency data by nonlinear regression and both products were compared by curve-fitting analysis (CFA, GraphPad Prism 6.05).

#### Active efflux

The MIC of TZP was determined without and with the non-selective efflux pump inhibitor phenylalanine-arginine β-naphthylamide (PAβN, Sigma-Aldrich, USA) that inhibits, among others, the wide-spectrum AcrAB-TolC pump [25]. A four-fold reduction in the MIC in the presence of PAβN (20 mg/L) was considered indicative of active efflux.

#### OmpF phenotype

The 30-μg cefoxitin disc method was used as a screening test for the reduction or loss of the porin OmpF [26, 27] comparing the inhibition zones of the parental ATCC *E*. *coli* 35218 and the isogenic 35218R strain. A 30% reduction in the diameter of the inhibition zone was considered a presumptive OmpF^¯^ phenotype.

#### Mutations in *bla*_*TEM-1*_

Total DNA of each *E*. *coli* strain was extracted by the boiling method. Briefly, one colony of the *E*. *coli* ATCC 35218, 35218R and 35218Δ was taken from an agar culture, it was dissolved in 10 mL of fresh Mueller Hinton Broth, and incubated at 37°C and 150 rpm in a water bath until it reached an OD_580_ of 0.6. After that, it was centrifuged at 10,000 rpm for 10 minutes, the supernatant was removed and the pellet was resuspended in 1 mL of PCR-grade water. Finally, it was immersed in boiling water during 15 minutes and then stored at -20°C until use. The PCR was done in a BIO-RAD C1000 thermal cycler with the primers Forward (5’-ATA AAA TTC TTG AAG ACG AAA-3’) and Reverse (5’-GAC AGT TAC CAA TGC TTA ATC A-3’), which amplify a fragment of ∼1000 bp encompassing the *bla*_*TEM-1*_ gene and its promoter [28], using the following mix: MgCl_2_ 2.5 mM, deoxynucleotides 0.2 mM, primers 0.7 mM each, Taq polymerase 0.05 U/μL (Promega, USA) and DNA sample 3 μL (final reaction volume: 50 μL). The thermal cycler PCR conditions were: 95°C for 3 minutes, then 35 cycles at 94°C (1 min), 50°C (1 min) and 72 °C (1 min), with a final extension of 2 minutes at 72°C. The PCR products, including the promoter and coding regions were sequenced by Macrogen (Seoul, South Korea) and then compared using Bioedit (version 7.2.5) with the published sequence of the *bla*_*TEM-1b*_ (GenBank accession number DQ058146.1) [29].

#### β-lactamase activity

The β-lactamase activity of *E*. *coli* strains ATCC 35218, 35218R and 35218Δ*bla* was determined by a nitrocefin (Becton-Dickinson, USA) degradation assay [30]. First, a total lysate of each strain was prepared taking one colony of *E*. *coli* from an agar culture, it was dissolved in 10 mL of fresh Mueller Hinton Broth (with the ATCC and R strains the medium contained 100 mg/L of ampicillin to ensure the expression of the β-lactamase), and incubated at 37°C and 150 rpm in a water bath until it reached an OD580 of 0.6. After that, it was centrifuged at 10,000 rpm for 10 minutes, the supernatant was removed and the pellet was resuspended in 5 mL of Tris-HCl-EDTA buffer (Qiagen Buffer AE, pH 7.0). Then, 40 μL of lysozyme (100 mg/mL) were added. The tubes were kept at room temperature for 60 minutes for the lysis to proceed. Finally, the lysates were stored at -70°C until use. The protein content of each lysate was measured by the Bradford method (BIO-RAD, USA). The amount of nitrocefin degraded per minute was determined by Beer’s equation with the change in absorbance at OD_486nm_ (Genesys 20 spectrophotometer, Thermo Scientific, USA) and a molar extinction coefficient of 20,500 M^-1^.cm^-1^, and the result was expressed as the nmol of nitrocefin degraded per minute per mg of protein [31]. The complete degradation profile was modelled with linear regression and compared by CFA (GraphPad Prism 6.05).

#### *bla*_*TEM-1*_ content

To determine if the increased β-lactamase activity was due to higher gene dose (i.e. higher plasmid copy number), the amount of the β-lactamase gene in the *E*. *coli* ATCC 35218 and 35218R strains was measured by quantitative real-time PCR, using primers for the *bla*_*TEM-1*_ gene located in the plasmid (Forward 5’ AAG CCA TAC CAA ACG ACG AG 3’ and Reverse 5’ TTG CCG GGA AGC TAG AGT AA 3’) and the single-copy *dxs* gene (d-1-deoxyxylulose 5-phosphate synthase), located in the chromosome (Forward 5’ CGA GAA ACT GGC GAT CCT TA 3’ and Reverse 5’ CTT CAT CAA GCG GTT TCA CA 3’). The GoTaq® qPCR Master Mix (Promega, USA) was used with a final primer concentration of 0.3 mM and the PCR conditions were: 95°C for 5 minutes, then 40 cycles at 95°C (10 seconds), 56°C for *dxs* or 58°C for blaTEM (10 seconds), and 72°C (10 seconds), with a final extension of 2 minutes at 72°C. The relative quantification was run in triplicate by the ΔΔCt method described by Lee et al. [32], using a real time thermal cycler (SmartCycler, Cepheid, USA) and the LinRegPCR software version 2014.5 [33].

#### *In vivo* growth rates

Using the data from *in vivo* experiments with pure and mixed inocula (described below), the growth rate (slope of the exponential phase) was estimated by fitting a modified Gompertz’ model to the time-growth data of untreated controls of the *E*. *coli* strains ATCC 35218, 35218R and 35218Δ*bla*. For comparative purposes, the growth rates of *S*. *aureus* GRP-0057 (a MSSA strain), *E*. *faecium* ATCC 51559 (a VRE strain), *P*. *aeruginosa* GRP-0019 (a wild-type strain) and *E*. *coli* SIG-1 (an ampicillin resistant strain) were estimated from previously obtained data (S1 Table). The equation, its parameters and interpretation are described in detail elsewhere [34].

### Pharmaceutical equivalence by liquid chromatography/mass spectrometry (LC/MS)

The concentration of the innovator and Farmalogica products was measured by liquid chromatography coupled to mass spectrometry (LC/MS). The pharmaceutical equivalence of generic TZP with respect to the innovator was determined by comparing the slopes and intercepts of standard curves of the freshly reconstituted products in sterile water (linear regression, Graphpad Prism 6.05). The assessment of the pharmaceutical forms by LC/MS included quantitative determination (SIM mode) of piperacillin and tazobactam, and qualitative analysis (SCAN mode) with an exploration range of 100–1000 daltons using an Agilent 1100 equipment coupled to a mass spectrometer electrospray ionization VL system. Chromatography was run through a C-18 column (one for each product) with a mixture of ammonium acetate and methanol (50:50 v/v ratio) as the mobile phase, at a flow rate of 0.5 mL/min. Mass spectrometry electrospray ionization was run in positive (H+) mode, monitoring eluents at 300 (tazobactam) and 518 daltons (piperacillin). The extraction time was optimized in order to obtain the fastest procedure without loss of analyte. The method allowed for simultaneous identification of piperacillin and tazobactam. The conditions of validation included selectivity, carry over test, matrix effects and extraction recoveries, linearity, accuracy, precision and stability. Each run was repeated at least twice.

### Mice

Murine-pathogen free (MPF) Swiss albino mice of the strain Udea:ICR(CD-2), bred at the University of Antioquia MPF vivarium were used in all kinetic and dynamic experiments. They were fed and watered *ad libitum*, housed at a maximum density of 7 animals per box within a 693 cm^2^ area in a One Cage System® (Lab Products, USA), and kept under controlled temperature (20°C and 25°C) and lightning conditions (12-hour day-night cycles). Inoculation in the thighs and euthanasia by cervical dislocation were done under isoflurane (Abbott, USA) inhaled anesthesia. Animals were randomly picked and allocated to treatment or control groups. The health condition of the mice was checked every day of the experiment and every 3 hours during the treatment phase (the last 24 hours). The following scale was used to classify the animals: 0: no signs of disease: active mouse, well groomed, alert, active; 1: mild signs of disease, as altered hair, slightly hunched posture with preserved mobility and response to stimuli; 2: moderate signs of disease, including squinted eyes, reduced mobility or reactivity, but able to reach water and food; 3: severe signs of disease, as great difficulty to reach water and food, dehydration (sunken eyes), reduced or no response to touch. If an animal reached phase 3 before the end of the experiment, it was humanely sacrificed. The study was reviewed and approved by the University of Antioquia Animal Experimentation Ethics Committee (session act No. 44, 2008) and complied with the national guidelines for biomedical research (Resolution 008430 of 1993 by the Colombian Health Minister, articles 87 to 93) and the ARRIVE guidelines (S1 File).

### Single-dose pharmacokinetics and bioequivalence

Three groups of 12 neutropenic female mice (neutropenia was induced by two doses of cyclophosphamide, see below), weighing 25±2 g and infected in the thighs with *E*. *coli* ATCC 35218, were allocated to one of these single subcutaneous (SC) doses of TZP: 640, 160 or 40 mg/kg. Each group was divided in 3 subgroups of 4 mice each. The first subgroup was sampled at 5, 45 and 90 minutes; the second at 15, 60 and 120 minutes; and the third group at 30, 75 and 150 minutes post-dose (all by retroorbital puncture). The samples were centrifuged to separate plasma and then frozen at -70°C. Piperacillin and tazobactam concentrations were determined by LC/MS as described above. Non-compartmental analysis (NCA) was used to estimate the AUC, elimination half-life, clearance and volume of distribution (PK package for R by T. Jaki, version 1.3–2). Bioequivalence was assessed comparing the clearances, volumes of distribution and areas-under-the-curve (AUC) by ANOVA (Graphpad Prism 6.05).

### Therapeutic equivalence of generic TZP in the neutropenic murine thigh infection model

With the Farmalogica generic, we performed 5 independent experiments comprising a total of 140 mice per product. In the case of the Procaps generic, two separate experiments were performed with 64 animals per product. The Farmionni and Vitalis generics were studied in a single experiment with 14 mice per product. In every case the innovator TZP (Wyeth) was included simultaneously. Female mice, weighing 23 to 27 g, were rendered neutropenic with two intraperitoneal doses of cyclophosphamide (150 and 100 mg/kg), injected 4 and 1 days before infection [35]. A volume of 0.1 mL of a log-phase culture containing ∼5.0 log_10_ CFU/mL of *E*. *coli* ATCC 35218 was injected in each thigh. Treatment began 2 hours after infection and lasted 24 hours. Seven groups of 2 to 5 animals received different total doses of TZP (innovator or generic) that ranged from no effect to maximal effect (80 to 5120 mg/kg per day divided q3h in 0.2 mL subcutaneous injections). At the end of treatment, mice were euthanized and their thighs were aseptically removed, homogenized, diluted, plated on MHA and incubated at 37°C for 18 hours. Antimicrobial effect was calculated by subtracting the CFU/g in the thighs of treated mice from untreated controls (a group of 2–5 mice mock-treated with 0.2 mL of sterile saline SC q3h). Dose-effect data were analyzed by nonlinear regression fitting Hill’s sigmoid model to estimate *E*_*max*_, *ED*_*50*_ and slope (*N*) for each TZP product (SigmaPlot 12.3), according to the following equation:

$$Effect=(-Emax*{Dose}^{N})/({ED}_{50}^{N}+{Dose}^{N})$$ (1)

and compared by CFA, using an extra-sum-of-squares F test (Graphpad Prism 6.05). Nonlinear regressions were assessed by the adjusted coefficient of determination (AdjR^2^), the standard error of estimate (S_y|x_), and the fulfillment of normality of residuals (by Shapiro-Wilk and D’Agostino-Pearson tests) and homoscedasticity (constant variance test). We checked for the absence of multicollinearity measuring the variance inflation factor (VIF), and considered free of multicollinearity any parameter with VIF<4 [36]. Each experiment was analyzed independently and jointly. In the latter case, the data were weighted by the inverse of the variance (1/SD^2^) to correct for heteroscedasticity. Normality of the residuals distribution was also assessed by the skewness and kurtosis: skewness quantifies how symmetrical the distribution is, with a value of zero for perfect symmetry and values >1 or <-1 indicating that the distribution is far from symmetrical. Kurtosis quantifies whether the shape of the distribution is Gaussian (kurtosis of zero, mesokurtic) or not: negative values (platykurtic) indicate a lower and broader central peak with shorter and thinner tails, while positive values (leptokurtic) indicate a higher and sharper peak with longer and fatter tails (Graphpad Prism 6.05) [37]. Accepting a 5% chance for a type I error, the basic design including treatment of 14 animals per product to compare innovator and 3 generic products confers 99% power to reject the null hypothesis, assuming that the magnitude of the difference in antifungal efficacy is ≥1.0 log_10_ CFU/g and the standard error of estimates is ≤0.5 log_10_ CFU/g (SigmaPlot 12.3).

One additional experiment with the same inoculum and doses (21 mice per product) was performed with the 35218Δ*bla* strain to test exclusively the therapeutic equivalence of the piperacillin component of TZP (innovator and Farmalogica generic), taking advantage of the fact that this strain lacks β-lactamase and tazobactam exerts no intrinsic effect on *E*. *coli*.

### Resistance enrichment *in vivo*

Considering that the frequency of spontaneous resistance of *E*. *coli* ATCC 35218 to TZP is very low (the resistance frequency to ≥16 mg/L of TZP is <10^−7.8^, as determined by the population-analysis profile), we prepared an inoculum of *E*. *coli* ATCC 35218 pre-seeded with *E*. *coli* 35218R in proportions from ∼0.3% to ∼6% following the method described by Negri et al. to study resistance selection in phenotypically heterogeneous bacterial populations [26]. Both strains were grown separately in broth to the log-phase (∼8 log_10_ CFU/mL), diluted and mixed before inoculation. The impact of TZP exposure on the resistance proportion was then assessed after 24 hours of treatment in neutropenic mice comparing the innovator and generic products.

Additionally, to test the impact of the *in vivo* growth of the resistant subpopulation on the effect of TZP, two different media were employed to prepare the inoculum: TSB, which, according to the size of the inoculum, resulted in net growth of *E*. *coli* 35218R in untreated controls up to ∼2 log_10_ CFU/g; and BHI, that yielded growths ≤ ∼1 log_10_ CFU/g. Using TSB, we performed two independent experiments with an initial resistance proportion of 0.5% comprising 30 mice per TZP product, and one experiment with a higher initial resistant proportion (6%) and 21 mice per product (the innovator and the generic Farmalogica). Using BHI, we carried out one experiment with a 0.8% resistant subpopulation with 21 mice per product (the innovator and Farmalogica) and one experiment with 0.3% resistant inoculum comprising 14 mice per product (the innovator and generic products Farmalogica and Procaps).

Treatment of all groups (innovator or generics, with 2 to 3 mice per dose) started two hours after infection using the same doses and schedules described for the therapeutic equivalence experiments. After 24 hours, thighs were plated on MHA and MHA with TZP (10 mg/L) to quantify total and resistant populations, respectively. The proportion of resistant bacteria was determined in each mouse by dividing the resistant population by the total population and the weighted mean (wMean) and standard deviation (wSD) were calculated for each dosing group, using the total number of bacteria per mouse as the weighting factor, with the following formulas (National Institutes of Standards and Technology, Information Technology Laboratory):

$${\bar{x}}_{w}=\frac{\sum_{i=1}^{n}w_{i}x_{i}}{\sum_{i=1}^{n}w_{i}}$$ (2)

$${SD}_{w}=\sqrt{\frac{\sum_{i=1}^{n}{w_{i}(x_{i}-{\bar{x}}_{w})}^{2}}{\frac{N^{\prime }-1}{N\prime }\sum_{i=1}^{n}w_{i}}}$$ (3)

Where *ẋ*_*w*_ is the weighted mean, *SD*_*w*_ the weighted standard deviation, *w*_*i*_ is the i^th^ weight of the i^th^ observation and N’ is the number of nonzero weights [38]. Innovator and generic resistance proportions at each dose were then compared by *t*-test with the Holm-Sidak post-hoc test to correct for the multiple comparisons (GraphPad Prism 6.05).

#### Time course of resistance enrichment *in vivo*

Neutropenic mice were infected with a mixed inoculum of *E*. *coli* ATCC 35218 and 35218R (0.5%). The dose of maximal enrichment of resistance in previous experiments with this inoculum was used (640 mg/kg/day), testing the Wyeth and Farmalogica products. Two mice from each treatment group and two infected but untreated animals were sacrificed at 6, 12, 18 and 24 hours. Thighs were homogenized and plated on MHA to quantify the total population and on MHA with TZP (10/1.25 mg/L) to measure the resistant subpopulation.

## Results

### *In vitro* activity of innovator and generic TZP

Innovator and generic had identical MIC (geometric mean) against *E*. *coli* ATCC 35218: 4/0.5 mg/L using the double dilution method recommended by CLSI, and 3/0.375 mg/L using the Jones-modified arithmetic dilution method. Against *E*. *coli* 35218R, both products had geometric mean of 32/4 mg/L under the double dilution method, while under Jones’s method it was 32/4 and 33.3/4.125 mg/L for Wyeth and Farmalogica, respectively (P = 0.37 by Student’s *t-*test). Against the 35218Δ*bla* strain, both products had the same piperacillin MIC (1 mg/L).

### Characterization of the 35218R strain

#### Susceptibility to other β-lactams

The 35218R strain was fully susceptible to cefoxitin, the third and fourth generation cephalosporins, aztreonam and the carbapenems (Table 1), ruling out the overproduction of constitutive AmpC β-lactamase as the responsible mechanism for TZP resistance [39].

**Table 1:** Susceptibility of *E*. *coli* 35218R to β-lactam antibiotics.

β-LACTAM	MIC (mg/L)
**Piperacillin/Tazobactam** *	32/4
**Cefoxitin**	≤ 4
**Ceftriaxone**	≤ 1
**Ceftazidime**	≤ 1
**Cefepime**	≤ 1
**Aztreonam**	≤ 1
**Imipenem**	≤ 1
**Meropenem**	≤ 0.25
**Ertapenem**	≤ 0.5

*Piperacillin-Tazobactam MIC was determined by broth microdilution, all the other antibiotics by Vitek® automatic system.

#### Population Analysis Profile (PAP)

**Fig 1:**
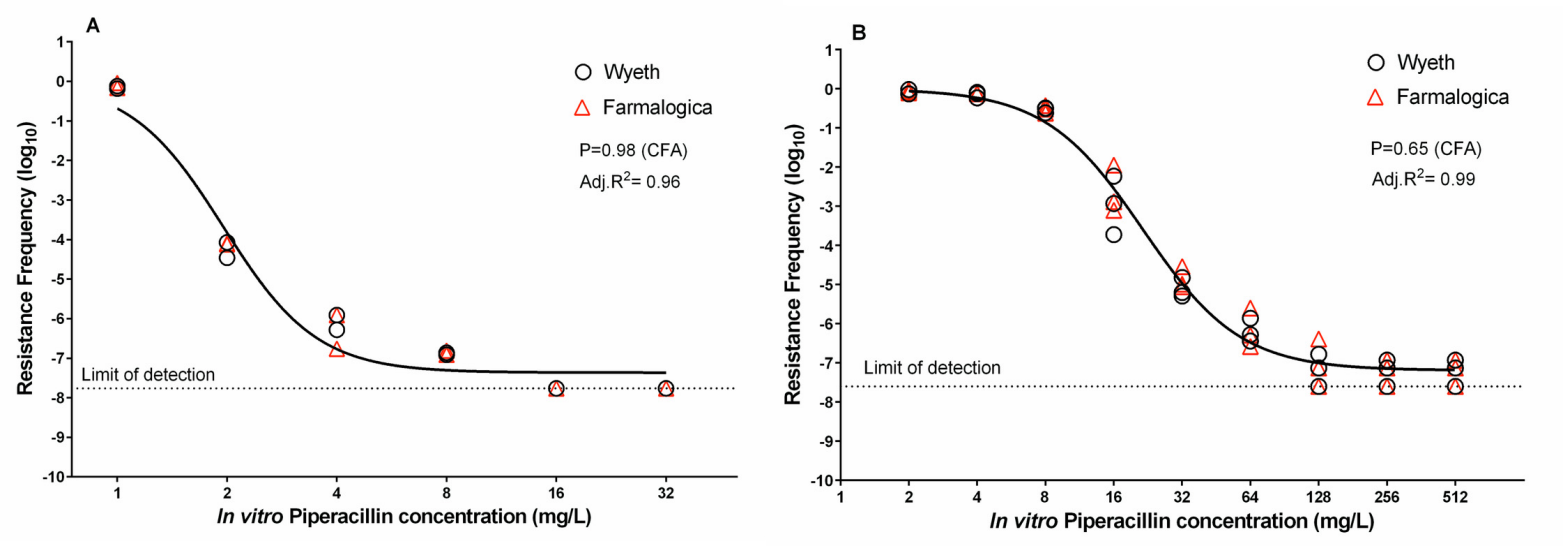
*In vitro* TZP population analysis profile (PAP) of *E*. *coli* ATCC 35218 and 35218R. TZP population analysis profile of *E*. *coli* ATCC 35218 (A) and *E*. *coli* 35218R (B). The resistance frequency data vs. TZP concentration was modelled using Hill’s equation and compared by CFA. There were no differences between innovator (Wyeth, black open circles) and generic (Farmalogica, red open triangles). Both data sets were better described by a single curve.

There were no differences between Wyeth and Farmalogica products in the PAP of *E*. *coli* ATCC 35218 and *E*. *coli* 35218R (Fig 1). The 35218R strain grew unrestrictedly up to 16 mg/L of antibiotic with a sharp decrease at 32 mg/L; a small subpopulation was able to grow up to 64 mg/L.

#### Active efflux

The MIC in the presence of PAβN was one dilution higher than the MIC without the efflux pump inhibitor: 64 vs. 32 mg/L, ruling out active efflux as the cause of resistance to TZP of the *E*. *coli* 35218R strain.

#### OmpF phenotype

The inhibition zones (mean ± SD) with the cefoxitin discs were 27.2 ± 0.1 mm for the ATCC strain and 27.9 ± 0.1 mm for the 35218R strain, indicating no loss of the OmpF porin as an enhancing mechanism of resistance to TZP.

#### Mutations of *bla*_*TEM-1*_

The PCR yielded an amplicon of ∼1000 bp with *E*. *coli* strains ATCC 35218 and 35218R, whereas the 38218Δ_*bla*_ strain was negative. The amplified sequence corresponded to *bla*_*TEM-1b*_ gene (Tn*2*) reported by Goussard and Courvalin [29, 40], and no mutations were found comparing the 35218R derivative with the parental ATCC strain. However, due to the specific location of the PCR product (very close to the end of the gene), the sequence of the 35218R strain could only be determined accurately up to the codon corresponding to amino acid 284 (alanine, according to Ambler numbering) [41], leaving out the last 6 residues. Notwithstanding, none of the 222 TEM β-lactamases currently reported at the Lahey Clinic classification [42], including the inhibitor-resistant TEM (IRTs), has mutations exclusively in those final residues (the only enzyme with a mutation in residue 289 is TEM-163 but it also has a substitution in residue 275). The promoter region corresponded to the classic *P3* promoter of *bla*_*TEM-1b*_ [40], and no mutations were found either when comparing the ATCC strain with 35218R (S1 Fig).

#### β-lactamase activity

**Fig 2:**
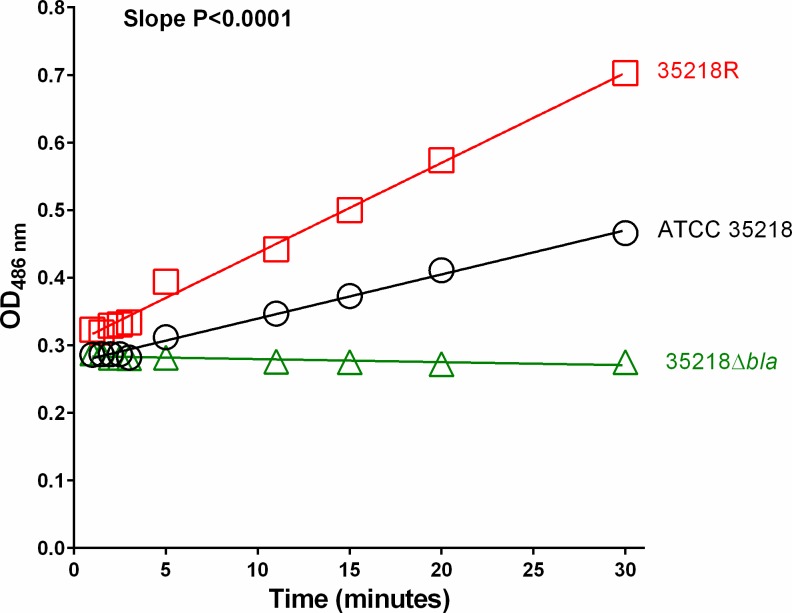
Nitrocefin degradation assay for *E*. *coli* ATCC 35218, 35218R and 35218Δ*bla*. Nitrocefin degradation by *E*. *coli* 35218R (red squares), *E*. *coli* ATCC 35218 (black circles) and *E*. *coli* 35218Δ*bla* (green triangles). The absorbance at 480 nm vs. time were modelled by linear regression and compared by curve fitting analysis (CFA). Strain 35218R (red squares and line) hydrolyzed nitrocefin more efficiently than the parental ATCC 35218 (black circles and line), while no hydrolysis occurred with the plasmid-cured isogenic strain 35218Δ*bla* (green diamonds and line), confirming β-lactamase hyperproduction by *E*. *coli* 35218R and negligible β-lactamase activity of *E*. *coli* 35218Δ*bla*. The P value from the slope comparison indicates that each group is described by an independent line.

Fig 2 illustrates the rate of nitrocefin degradation by the three strains. The 35218R strain degraded nitrocefin at a higher rate than the parental ATCC 35218 strain (P of slope <0.0001 by CFA). The cured strain (38218Δ*bla*) had a negligible effect on nitrocefin. After normalizing by the protein content, the rates of nitrocefin degradation were 280, 120 and 16 nmol per minute per mg of protein, for 35218R, ATCC 35218 and 35218Δ*bla* strains respectively, confirming that hyperproduction of β-lactamase, not a new mutation, was the responsible mechanism for resistance of *E*. *coli* 35218R.

#### *bla*_*TEM-1*_ content

The ΔΔCt was 4.62 and the mean efficiency of the qPCR was 1.675. The 35218R strain had 10.75 ± 0.75 (mean ± standard error) more *bla*_*TEM-1*_ than the parental ATCC 35218 strain, confirming that increased beta-lactamase activity was due to higher gene dose, in agreement with previous reports indicating that hyperproducer strains have on average 22 plasmid copies in contrast to 2.2 copies in normally producing strains [31].

#### *In vivo* growth rate

The growth rates of the parental (ATCC 35218) and resistant (35218R) strains were the same when using each one in a pure inoculum (0.64 ± 0.16 and 0.62 ± 0.08 h^-1^, respectively; P = 0.38 for slopes comparison by CFA), but the 24 h total growth of the resistant strain was almost 1 log_10_ lower. Such handicap is explained by the fitness cost of resistance and it was overcome after curing the plasmid (total growth of 5.29 ± 0.16 and 4.39 ± 0.08 log_10_ CFU/g for the cured Δ*bla* and 35218R strains, respectively). The individual growth rates of our *E*. *coli* strains were also similar to those of other susceptible and resistant strains of clinical origin: *S*. *aureus* GRP-0057, *E*. *faecium* ATCC 51559, *P*. *aeruginosa* GRP-0019 and *E*. *coli* SIG-1 (S1 Table).

### Pharmaceutical equivalence

**Fig 3:**
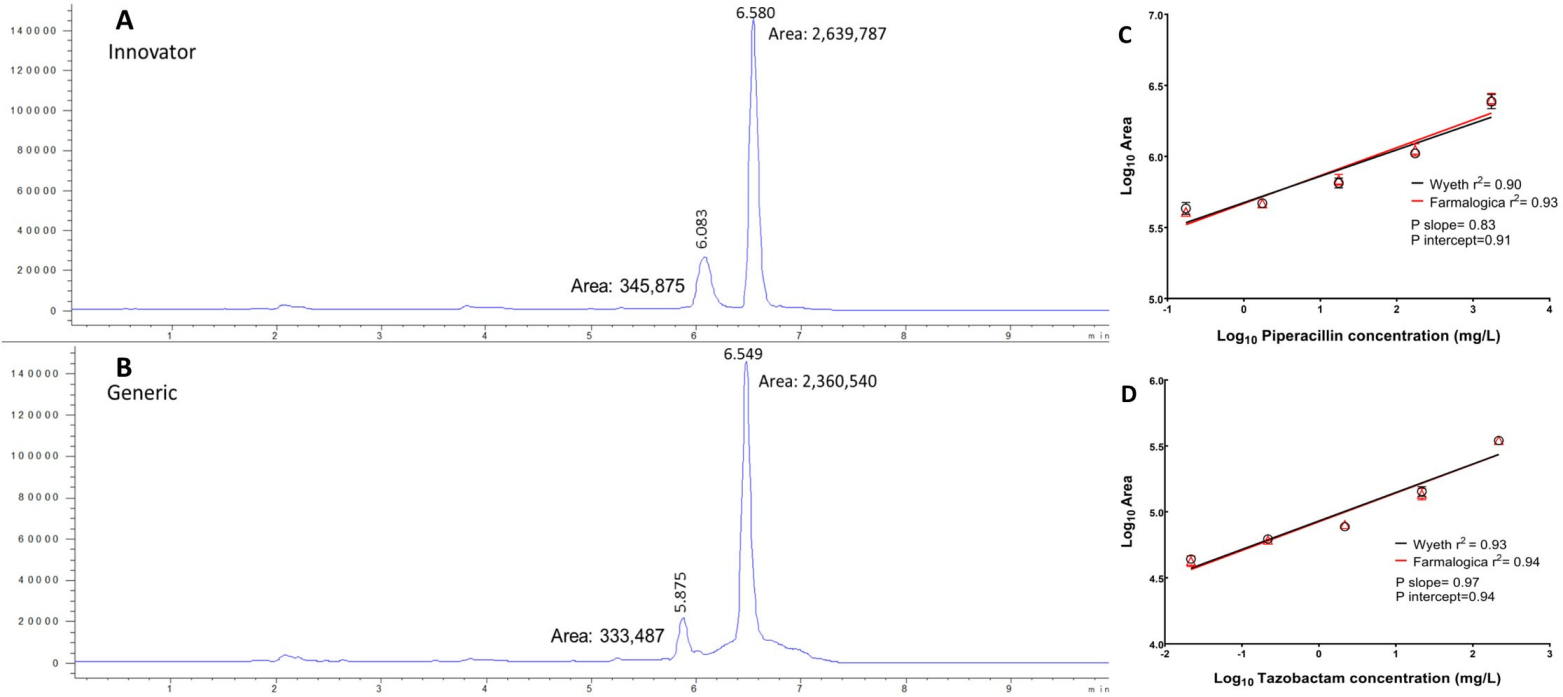
LC/MS comparison and pharmaceutical equivalence of innovator (Wyeth) and generic (Farmalogica) TZP. Panels A and B display the chromatograms of freshly reconstituted innovator and generic TZP, at the highest tested concentration (1740 mg/L of piperacillin and 217.5 mg/L of tazobactam). The small peak corresponds to tazobactam and the highest peak to piperacillin. The retention times, peak magnitudes and areas of both products were very similar. Panels C and D show the standard curves of piperacillin and tazobactam. The regression analysis comparing slopes and intercepts indicated that a single curve described both innovator and generic products, which are therefore pharmaceutically equivalent.

Calibration curves of the freshly reconstituted products were linear over the range of 0.17–1740 mg/L for piperacillin and 0.02–217.5 mg/L for tazobactam. The retention times were 6.580 min and 6.549 for innovator and generic piperacillin, and 6.083 and 5.875 min for innovator and generic tazobactam, respectively; the intraday and interday variation was <1%. Recovery was 99.8–103.4% for piperacillin and 98.7–103.5% for tazobactam. Despite minor differences in the areas of both products, the standard curves for the piperacillin and tazobactam components of TZP Wyeth and Farmalogica were overlaid, without differences in slopes (P = 0.83 for piperacillin and P = 0.97 for tazobactam) or intercepts (P = 0.91 for piperacillin and P = 0.94 for tazobactam), indicating that the generic product was pharmaceutically equivalent to the innovator. Fig 3 displays the LC profiles of Wyeth (panel A) and Farmalogica (panel B) products at the highest tested concentration showing the respective peaks for both piperacillin and tazobactam and, in panels C and D, the component’s standard curves, demonstrating that generic and innovator belonged to the same population (overlaid lines).

### Qualitative analysis by LC/MS

**Fig 4:**
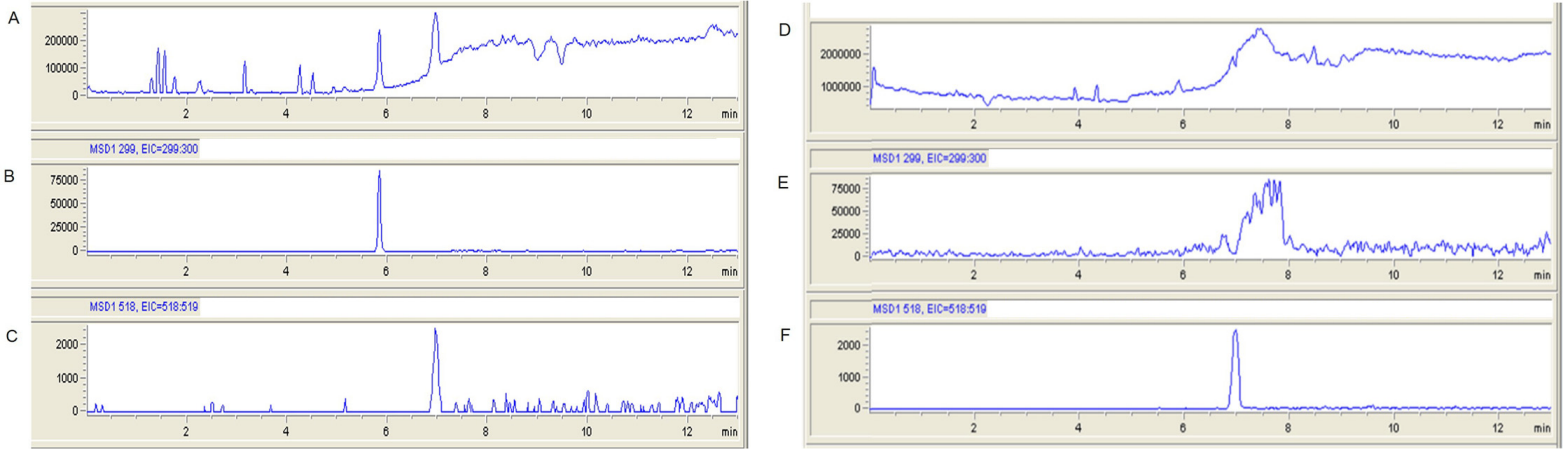
LC/MS analysis of innovator (Wyeth) and generic (Farmalogica) TZP. LC/MS analysis of TZP of innovator (Wyeth, panels A, B and C) and generic (Farmalogica, panels D, E and F) TZP from mouse serum 2 hours after the 640 mg/kg dose. Panels A and D correspond to the complete spectrogram along a 13-minute run, Panels B and E to the specific spectrogram of tazobactam (299:300 Da) and Panels C and F to the specific spectrogram of piperacillin (518:519 Da). The spectrum of the tazobactam component differed markedly between Farmalogica (Panel E) and Wyeth (Panel B). In the generic, the tazobactam peak appears unstable because there are compounds with the same molecular mass but different retention times, while in the innovator the tazobactam peak is neatly defined. It suggests product isomer heterogeneity in the tazobactam component of the Farmalogica product.

The most important structures were present in both, generic and innovator products: parent ions piperacillin (518.2 Da) and tazobactam (299.1 Da); daughter ions piperacillin (359 Da and 143 Da) and tazobactam (138 Da and 254 Da). Fig 4 displays the spectral features in the scan exploration of samples from the mouse PK (Panels A, B, C for Wyeth, and D, E, F for Farmalogica). With the innovator, a clear piperacillin peak (518 Da) was observed at 6.8 minutes and a clear tazobactam peak (299 Da) at 5.9 minutes. With the generic, a defined piperacillin peak was also observed at the same time than the innovator but, in the case of tazobactam, a series of irregular peaks corresponding to masses between 299 and 300 Da appeared between 6 and 8 minutes. This unstable behavior suggests product heterogeneity, possibly indicating different configurations (isomers) of the triazole and carboxyl groups of tazobactam [43].

### Single-dose pharmacokinetics and bioequivalence

Table 2 presents the estimate of the PK parameters clearance and volume of distribution (mean and standard error) and the AUC_0-∞_ from the three doses tested. Regarding bioequivalence between generic and innovator products, there were no significant differences in clearances and volumes of distribution with piperacillin nor tazobactam, and as expected, there were no differences either in the AUC from the three doses tested. Of note, the generic that failed therapeutic equivalence (Farmalogica) passed the “bioequivalence” test as well as the generic that passed therapeutic equivalence (Procaps).

**Table 2:** Pharmacokinetics of Piperacillin and Tazobactam.

Parameter	PIPERACILLIN				TAZOBACTAM			
	Wyeth	Farmalogica	Procaps	P (ANOVA)	Wyeth	Farmalogica	Procaps	P (ANOVA)
**Clearance (L/h)**	0.114 (0.011)	0.111 (0.008)	0.110 (0.012)	0.978	0.064 (0.004)	0.068 (0.01)	0.066 (0.006)	0.917
**Volume of distribution (L)**	0.071 (0.010)	0.069 (0.010)	0.073 (0.010)	0.960	0.040 (0.006)	0.036 (0.003)	0.036 (0.002)	0.731
**AUC**_**0-∞**_ **(mg.h/L) 640–80 mg/kg**	140.1 (3.52)	139.4 (4.65)	132.6 (2.65)	0.349	32.6 (0.78)	34.4 (0.99)	34.5 (0.95)	0.079
**AUC**_**0-∞**_ **(mg.h/L) 160–20 mg/kg**	30.2 (0.46)	32.4 (0.79)	32.2 (0.64)	0.098	8.29 (0.44)	8.55 (0.41)	8.01 (0.36)	0.653
**AUC**_**0-∞**_ **(mg.h/L) 40–5 mg/kg**	10.6 (0.37)	10.5 (0.63)	11.3 (0.53)	0.512	1.77 (0.31)	1.40 (0.18)	1.57 (0.20)	0.569

The parameter estimates are expressed as mean (and standard error in parentheses). The AUC_0-∞_ corresponds to the area under the concentration-time curve from time zero to infinity yielded by the 3-dose levels of TZP (8:1 piperacillin:tazobactam ratio), each as a single subcutaneous injection.

### Therapeutic equivalence against *E*. *coli* ATCC 35218 and *E*. *coli* 35218Δ*bla*

**Fig 5:**
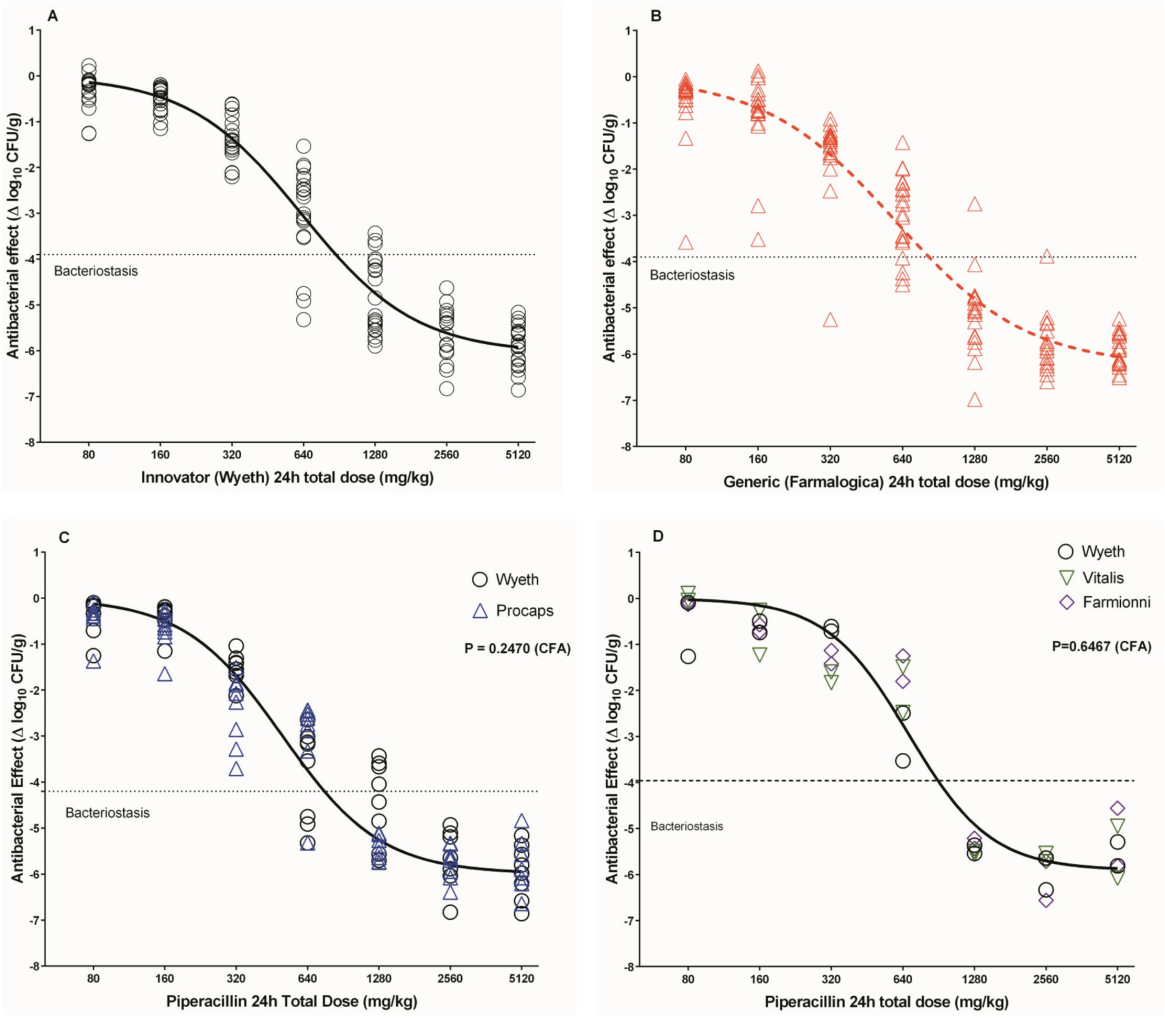
*In vivo* pharmacodynamics of innovator and generic TZP against *E*. *coli* ATCC 35218 in the NMTIM. A. Pharmacodynamics of innovator (Wyeth) TZP from 5 independent experiments. The graph shows the dose-response curve of the innovator (black open circles) with a valid regression. B. Pharmacodynamics of generic TZP (Farmalogica) from 5 independent experiments. The regression is invalid for failing the normality of the residuals assumption (the dotted curve implies a faulty fit). Compared with the innovator (panel A), the dose-response relationship of Farmalogica is erratic and the data more dispersed. C. Therapeutic equivalence of generic TZP (Procaps, blue open triangles) with the innovator (Wyeth, black open circles), combining data from 2 independent experiments. Both products yielded valid regressions and the CFA demonstrated that a single curve described better the two datasets, indicating that this generic TZP was therapeutically equivalent to the innovator. D. Therapeutic equivalence of two generics of TZP (Vitalis, green open triangles; and Farmionni, purple open triangles) with the innovator (Wyeth, black open circles). All three products generics yielded valid regressions and the CFA demonstrated that a single curve described better the three datasets, indicating that both generics were therapeutically equivalent to the innovator.

The five experiments comparing TZP Wyeth and Farmalogica were analyzed individually and in combination. In contrast with the innovator, the generic failed the normality of the residuals test from the first experiment (with 7 doses and 2 animals per dose). To rule out insufficient sampling as the cause of non-normality, the number of animals was increased to 3 and then to 5 per dose, but the normality test for Farmalogica data kept failing. Combining all the data we had 140 mice per product, but the generic Farmalogica still failed normality and displayed an erratic PD behavior not seen in the normally distributed data from the mice treated with the innovator (Table 3 and panels A and B of Fig 5). The kurtosis of Farmalogica distribution was near 4, indicating a markedly leptokurtic curve. The residuals plot is presented in S2 Fig to further illustrate the generic’s non-normal behavior. As the lack of normality invalidates the fitting of Hill’s model by least-squares nonlinear regression, the magnitudes of the PD parameters of generic and innovator could not be compared statistically. Therefore, the generic was considered nonequivalent exclusively on the basis of its lack of normality in the dose-effect nonlinear regression, and we proceeded to test if this nonequivalence had any impact on the enrichment of the resistant subpopulation of *E*. *coli*. In the experiments with the Procaps generic, the residuals had a normal distribution, all the parameters were significant and the AdjR^2^ was 0.945 for Wyeth and 0.959 for Procaps. The global CFA indicated that a single curve described best the two products (P = 0.30), and thus this generic demonstrated its therapeutic equivalence to the innovator (Table 3 and panel C of Fig 5). The Farmionni and Vitalis products yielded valid regressions and were also therapeutically equivalent to the innovator (Table 3 and panel D of Fig 5).

**Table 3:** Pharmacodynamic parameters (PDP) and model diagnostics of innovator (Wyeth) and four generics of TZP against *E*. *coli* ATCC 35218.

Nonlinear regression	Wyeth	Farmalogica*	Procaps	Vitalis	Farmionni
***E***_***max***_ **(log**_**10**_ **CFU/g)**	6.08 (0.12)	--	6.02 (0.17)	5.98 (0.53)	5.77 (0.40)
***ED***_***50***_ **(mg/kg day)**	634.5 (39.5)	--	435.7 (35.9)	653.7 (120.1)	777.8 (89.9)
***N***	1.75 (0.10)	--	1.90 (0.18)	1.96 (0.56)	3.94 (1.46)
**AdjR**^**2**^	0.9614	--	0.9590	0.9065	0.9108
**S**_**y|x**_ **(log**_**10**_ **CFU/g)**	0.9988	--	1.0983	0.7338	0.7378
**Normality test**	Passed	Failed	Passed	Passed	Passed
**Skewness**	-0.4745	-0.8629	-0.4392	0.7669	0.6204
**Kurtosis**	-0.2707	3.756	-0.2964	0.4187	0.9359

The PDP are shown as mean (standard error).

*PDP of TZP Farmalogica are not comparable (therefore excluded) because the dose-effect relationship of this product did not pass the normality test.

**Fig 6:**
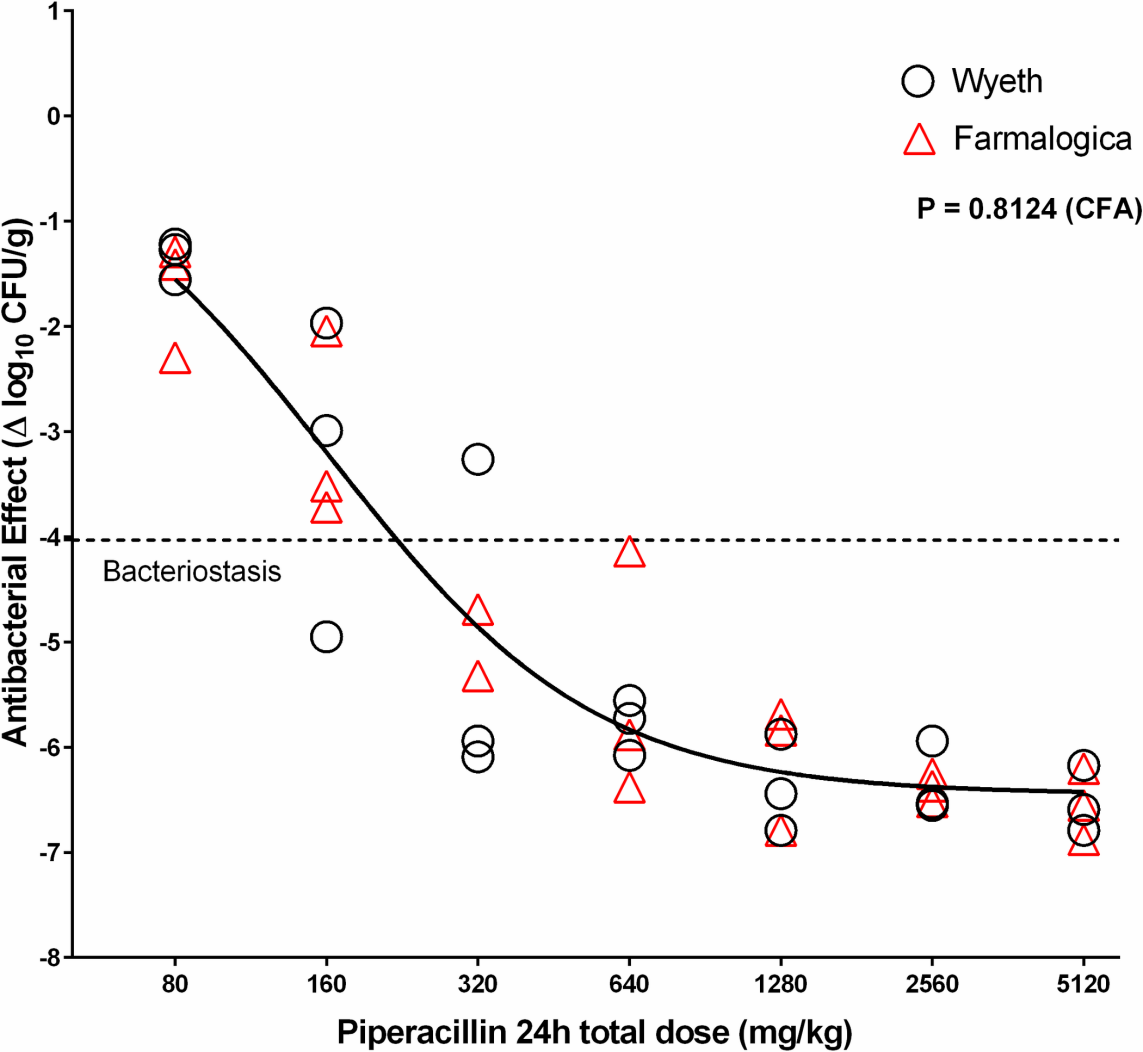
*In vivo* pharmacodynamics of innovator (Wyeth) and generic (Farmalogica) TZP against *E*. *coli* 35218Δ*bla*. Therapeutic equivalence of generic TZP (Farmalogica) with the innovator (Wyeth) against *E*. *coli* 35218Δ*bla*, a strain without β-lactamase (plasmid cured). After treatment of mice infected with the cured strain, TZP Farmalogica had a valid nonlinear regression fulfilling all the assumptions with highly significant PD parameters. Both products were described by a single curve, indicating that the generic is therapeutically equivalent to the innovator. As the strain has no β-lactamase, the effect is solely from the piperacillin component of TZP and indicates that the nonequivalence found against *E*. *coli* ATCC 35218 is due to tazobactam.

In order to test the effect of piperacillin without the influence of tazobactam, we infected the mice with *E*. *coli* 35218Δ*bla*, a plasmid-cured strain that does not have the TEM-1 β-lactamase. In this case, both Wyeth and Farmalogica generated valid regressions, fulfilling the assumptions of normality and homoscedasticity, and a single curve described best the PD behavior (global CFA P = 0.8), indicating that the piperacillin component of the generic was therapeutically equivalent. This result also suggests that the lack of normality against *E*. *coli* ATCC 35218, in which we based our designation of nonequivalence for TZP Farmalogica, was due to the tazobactam component (Table 4 and Fig 6).

**Table 4:** Pharmacodynamic parameters (PDP) and model diagnostics of Wyeth and Farmalogica TZP against *E*. *coli* 35218Δ*bla*.

Nonlinear Regression	Wyeth	Farmalogica
***E***_***max***_ **in log**_**10**_ **CFU/g**	6.43 (0.28)	6.49 (0.27)
***ED***_***50***_ **in mg/kg per day**	158.5 (20.0)	166.1 (20.4)
***N***	1.85 (0.42)	1.44 (0.27)
**AdjR**^**2**^	0.8467	0.8906
**S**_**y|x**_ **in log**_**10**_ **CFU/g**	0.7793	0.6075
**Normality test**	Passed	Passed
**Constant variance**	Passed	Passed

PDP are shown as mean (standard error).

### Resistance enrichment

There was a sharp difference between the innovator and the generic Farmalogica regarding the selection of resistance, with the later exhibiting an “inverted U” dose-response relationship, with minimal enrichment at the highest and lowest doses and a peak of maximal selection at the middle ones, ranging from 320 to 1280 mg/kg per day, according to the inoculum.

**Fig 7:**
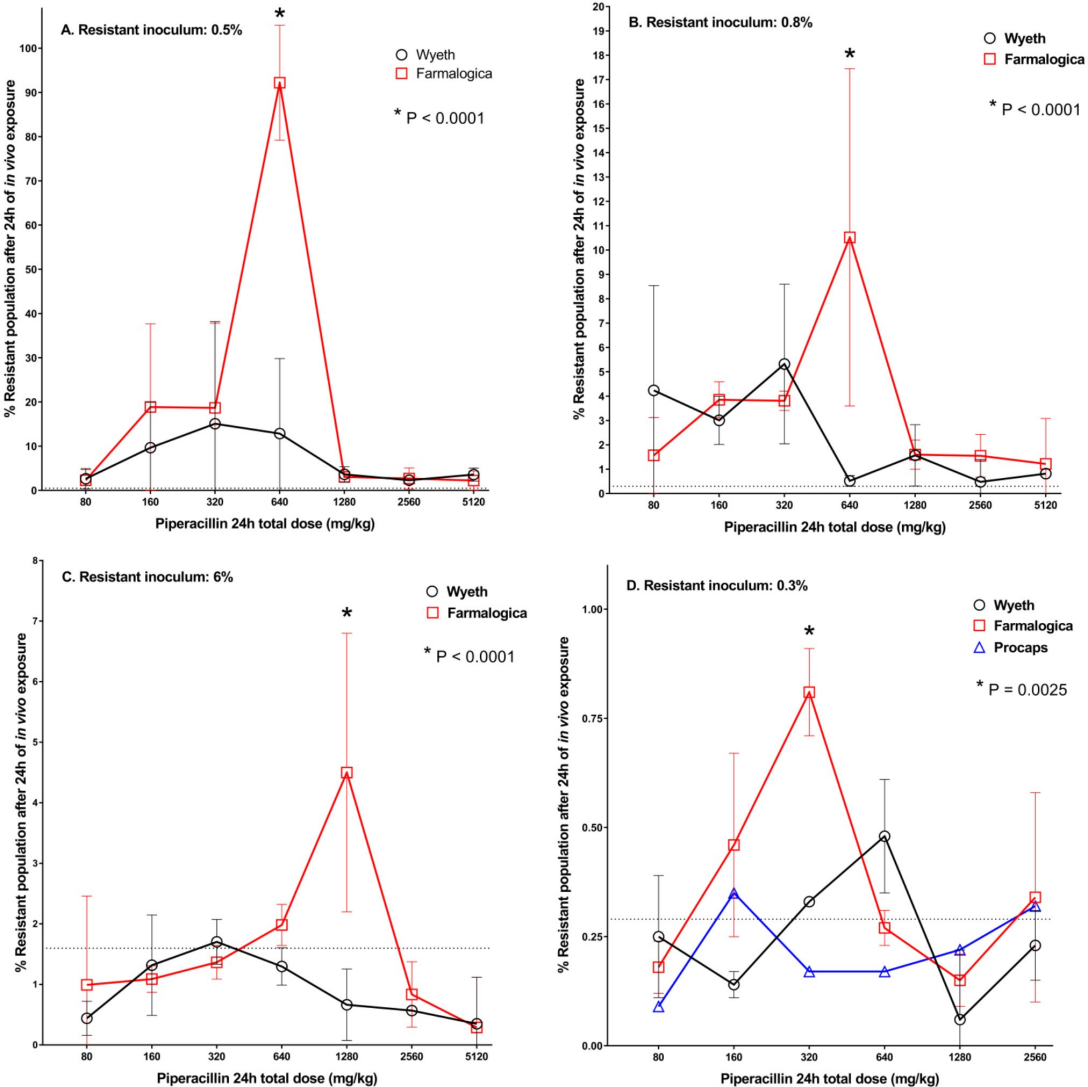
Resistance proportion after *in vivo* exposure of a mixed *E*. *coli* population to innovator (Wyeth) and generic (Farmalogica) TZP. A. Resistant inoculum of 0.5%. At the start of treatment (2 hours after inoculation) the proportion of resistance had dropped to 0.25% (dotted line) and the net growth of the resistant subpopulation in untreated controls reached 2 log_10_ CFU/g. The generic significantly enriched the resistant subpopulation at 640 mg/kg per day (P<0.0001), without differences at the other doses, in an “inverted U” pattern. The graph shows the weighted mean and standard deviation (wMean and wSD) from 2 independent experiments comprising 5 mice per product per dose. B. Resistant inoculum of 0.8%. At the start of treatment the proportion of resistance had dropped to 0.3% (dotted line) and the net growth of the resistant subpopulation in untreated controls was 0.8 log_10_ CFU/g. The generic significantly enriched the resistant subpopulation at 640 mg/kg per day (P<0.0001), without differences at the other doses. The graph shows 3 mice per product per dose. C. Resistant inoculum of 6%. At the start of treatment the proportion of resistance dropped to 1.6% (dotted line) and the net growth of the resistant subpopulation in untreated controls was 0.5 log_10_ CFU/g. The generic significantly enriched the resistant subpopulation at 1280 mg/kg per day (P<0.0001), without differences at the other doses. The graph shows 3 mice per product per dose. D. Resistant inoculum of 0.3%. At the start of treatment the proportion of resistance declined to 0.29% (dotted line) and the net growth of the resistant subpopulation in untreated controls was 1.2 log_10_ CFU/g. There were no differences between the innovator and the generic Procaps at any dose. The generic Farmalogica, on the other hand, significantly enriched the resistant subpopulation at 320 mg/kg per day (P = 0.0025). The apparent increase in resistance with the innovator at 640 mg/kg per day was not statistically significant after the post-hoc test. The graph shows 2 mice per product per dose.

In the two experiments using TSB with an initial resistant inoculum of 0.5% (panel A of Fig 7 and S2 Table), the Farmalogica generic significantly enriched resistance at 640 mg/kg per day, with the resistant cells reaching 92% of the population in contrast to 13% with the innovator (P<0.0001). In the experiment with a resistant inoculum of 0.8%, but using BHI instead of TSB to grow the resistant strain, the generic Farmalogica significantly enriched resistance at 640 mg/kg per day, although to a smaller extent compared with the first experiment: 0.5% vs. 10.5% for innovator and generic, respectively, P<0.0001 (panel B of Fig 7 and S3 Table). When the resistant inoculum was increased to 6% (using TSB), the generic Farmalogica significantly enriched resistance at 1280 mg/kg per day: 0.7% vs. 4.5% for generic and innovator, respectively, P<0.0001 (panel C of Fig 7 and S4 Table). In the last resistance-enrichment experiment, using BHI and an initial proportion of resistance of 0.3%, the generic Farmalogica significantly enriched resistance at 320 mg/kg per day: 0.33% vs 0.81% for innovator and generic, respectively, P = 0.002 (panel D of Fig 7 and S5 Table), whereas the equivalent generic Procaps showed no difference with the innovator at any dose, in agreement with the data obtained with ciprofloxacin equivalent generics [21].

Noticeably, the maximal magnitude of enrichment was related to the net growth of the resistant subpopulation in controls, reaching values with the generic from 50–90% when growth was 1.8–2.2 log_10_ CFU/g (using TSB as growth medium for the inoculum) and values from 1–10% when growth ranged from 0.5–1.2 log_10_ CFU/g (using BHI as growth broth). The difference in growth might be related to the impact of the medium composition during the inoculum preparation, but more research is necessary to elucidate this point.

#### Time course of resistance enrichment

**Fig 8:**
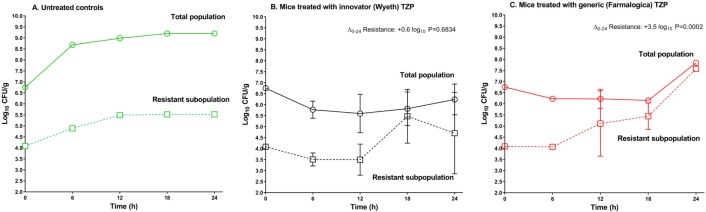
Time course of resistance enrichment *in vivo* by innovator (Wyeth) and generic (Farmalogica) TZP at 640 mg/kg per day. Population dynamics of the total mixed population (circles and continuous lines) and the resistant subpopulation (squares and dotted lines), after *in vivo* exposure in neutropenic mice to 640 mg/kg per day. Two mice were sacrificed at each time-point except for the last one (24h) where 3 animals were used. A. Untreated controls (green), the total population grew 2.44 log_10_ CFU/g, and the resistant one 1.44 log_10_ CFU/g. B. Innovator TZP treatment group (black), the resistant subpopulation started to expand after the 12^th^ hour, at the end of the experiment it had increased in average 4-times compared with initial numbers, a nonsignificant difference (P = 0.6834). C. Generic Farmalogica TZP treatment group (red), the resistant subpopulation began to expand after 6 hours, increasing in average 3162-fold at the end of therapy (P = 0.0002). Resistance enrichment with the nonequivalent generic was at least 800-times higher than with the innovator. The symbols represent the mean bacterial burden and the error bars the standard deviation (log_10_ CFU/g).

In this experiment the total inoculum had 6.61 log_10_ CFU/mL, and 4.26 log_10_ CFU/mL of them (0.45%) were resistant cells. The total population in the control group reached 9.2 log_10_ CFU/g at the end of the experiment with a resistant subpopulation of 5.55 log_10_ CFU/g (net growth from 0h to 24h: 1.45 log_10_ CFU/g). In the innovator-treated group, the total population changed from 6.8 to 6.2 log_10_ CFU/g and the resistant subpopulation increased from 4.1 to 4.7 log_10_ CFU/g (Δ +0.6, a statistically nonsignificant 3.98-fold increment, P = 0.6834). With the generic Farmalogica, the total population increased from 6.8 to 7.8 log_10_ CFU/g and the resistant cells from 4.1 to 7.6 log_10_ CFU/g (Δ +3.5 log_10_, a statistically significant 3162-fold increment, P = 0.0002). Thus, the resistance enrichment seen with the generic was ∼800 times higher than the innovator. The percentage of resistance (weighted mean) along the 24 hour period of the whole experiment was 20% (weighted SD of 26%) in the innovator group and 51% (weighted SD of 16%) in the generic group, a significant difference by *t*-test (P = 0.0027). Additionally, the resistant subpopulation began to grow after the sixth hour of treatment with the generic and after the twelfth hour with the innovator, and the steepest increase was observed from hours 18 to 24 (Fig 8).

## Discussion

We have previously demonstrated that the pharmaceutical equivalence of generic products of oxacillin, vancomycin, gentamicin and meropenem, among other antibiotics, does not assure their therapeutic equivalence in the validated neutropenic thigh infection model [11–14, 16]. In those studies, we developed a statistical framework to assess *in vivo* equivalence by fitting dose-response data to Hill’s sigmoid *E*_*max*_ model by ordinary least-squares nonlinear regression (NLR) to estimate primary (*E*_*max*_, *ED*_*50*_, *N*) and secondary PD parameters (Bacteriostatic Dose or *BD*, 1-log_10_ kill dose or *1LKD*). The dose-response curves are then compared by global curve fitting analysis (CFA) by an extra-sum-of-squares *F*-test to determine if a single curve describes all data (equivalence) or each data set is best described by an individual curve (nonequivalence). This analysis also allows the comparison of individual PD parameters to detect specific differences between generic and innovator *in vivo*, in terms of efficacy (*E*_*max*_) or potency (*ED*_*50*_, *BD*, *1LKD*).

Several lines of evidence indicate that therapeutic nonequivalence of generic antibiotics determined in the NMTIM under Hill’s model is biologically and clinically relevant, and has an impact on outcomes such as dissemination to distant organs [13], therapeutic failure and increased mortality [44–46] and, even more worrisome, on resistance. We demonstrated before that generics of vancomycin failing therapeutic equivalence significantly enriched the less susceptible subpopulations (able to grow in up to 3 mg/L of the antibiotic) of *S*. *aureus* after 12 days of *in vivo* exposure, compared with the innovator that, in fact, reduced them [18]. In contrast, a therapeutically equivalent generic of ciprofloxacin was identical to the innovator when selecting resistant mutants of *P*. *aeruginosa* after 7 days of exposure to clinically achievable concentrations in the hollow fiber PD system [21]. Thus, the data indicate that therapeutic equivalence in terms of antibacterial activity entails equivalence in resistance outcomes.

Here we present a new case of therapeutic nonequivalence, this time expressed as a non-normal dose-response relationship that precludes the fitting of Hill’s model by least-squares nonlinear regression and subsequent statistical comparison with the innovator. The fact that the innovator’s effect is predictable and follows a Gaussian distribution (effect residuals with a mean of zero at each dose and 95% of data within two standard deviations), while the generic product does not despite using a sufficiently large sample and appropriate design, indicates an erratic PD response that deserves an explanation and could have measurable consequences such as enrichment of resistant cells, as was in fact demonstrated.

The generic TZP Farmalogica displayed therapeutic equivalence against the β-lactamase nonproducing strain *E*. *coli* 35218Δ*bla*, but failed against its parent strain that produces TEM-1 β-lactamase, *E*. *coli* ATCC 35218, indicating that tazobactam is the component responsible for nonequivalence: against *E*. *coli* 35218Δ*bla*, the observed effect depended solely on piperacillin, whereas in the case of *E*. *coli* ATCC 35218 it was the result of the interaction of both compounds, in which tazobactam must prevent the degradation of piperacillin so it can act upon the PBPs. Recent studies indicate that the percentage of time that the free concentrations of the inhibitor are above a certain threshold (*f*T_>threshold_) is the PD index driving the efficacy of β-lactamase inhibitor combinations as ceftolozane-tazobactam [47] and piperacillin-tazobactam [48]. The LC/MS analysis demonstrated that the tazobactam component of the generic Farmalogica has heterogeneity problems in terms of isomers that affect its interaction with the β-lactamase (see below); it explains why it failed to achieve the PD target necessary for efficacy and displays an erratic, unpredictable profile *in vivo* that enriched the resistant subpopulation of *E*. *coli* characterized by β-lactamase hyperproduction (*E*. *coli* 35218R).

A potential limitation to consider is that we used single lots of each generic. However, it does not invalidate the findings because the generic that failed therapeutic equivalence did pass all the “quality” tests required by drug regulatory agencies (DRA), including pharmaceutical equivalence, bioequivalence, and *in vitro* potency. As a general principle, when problems arise with the quality of generic medicines, all these tests fail (not only therapeutic equivalence), as demonstrated with generics of oxacillin [12]. Although our protocols do check for quality, our research problem is the DRA assumption that pharmaceutical equivalence implies therapeutic equivalence. Within that endeavor, we only study generics of “good quality” that are being used every day to treat sick patients because have been tested and found “bioequivalent” by DRA. And most important, our study of gentamicin generics indicated that the problem of nonequivalence is batch-independent, with no difference in the frequency of nonequivalence between the products in which the same lot or different ones were tested [13].

Bacterial resistance to antibiotics is one of the best documented examples of contemporary biological evolution [5], and the methodology of experimentally mimicking heterogeneous bacterial populations has been used to study resistance selection under antibiotic pressure. The group of Baquero and Levin used cefotaxime to treat a TEM-1 positive *E*. *coli* population containing a 1% of TEM-12 producers cells (a β-lactamase with a single amino acid substitution that confers a slightly higher MIC), demonstrating *in vitro* and *in vivo* amplification of the less susceptible subpopulation (in this case with a minor selective advantage in face of antibiotic exposure) within a concentration selection window [26]. These subpopulations may then acquire additional determinants that lead to clinically significant resistance (e.g. porin loss or additional mutations). A similar approach using mixed bacterial populations to study resistance selection has been employed with *E*. *coli* [49], *Streptococcus pneumoniae* [50–52], *Staphylococcus aureus* [53] and *Mycobacterium tuberculosis* [54].

The difference between innovator and generic could only be seen in the animal model, suggesting that the *in vitro* tests are not sensitive to detect subtle but pharmacodynamically significant differences between products [55]. It is well-known that tazobactam is a difficult-to-produce drug because of the unwanted formation of useless and hard-to-separate isomers like benzhydryl 2-((R)-2(2-(benzo[d]thiazol-2-yl)disulfanyl)-4-oxoacetidin-1-yl)-3-methylbut-2-enoate, during the process of synthesis [56]; also by the presence in the final product of another inactive and difficult-to-purify isomer with R configuration in the C-3 atom, instead of the S configuration of tazobactam [57], and by problems of hygroscopicity and instability of the lyophilized form of tazobactam-sodium that have led to the patenting of new crystallization strategies [58] or new salts like tazobactam-arginine [59]. In addition, tazobactam suffers spontaneous hydrolysis in solution at 37°C, and undergoes hepatic metabolism to an inactive compound (metabolite M_1_) [60]. In the case of this particular Farmalogica generic, we found that the product displayed heterogeneity regarding isomer composition, as strongly suggested by the wide and irregular peak observed in the LC/MS analysis, and that it led to the enrichment of a harder-to-treat subpopulation overproducing β-lactamase.

Finally, some considerations regarding generic antibiotics nonequivalence characterized by the violation of the normality assumption of least-squares regression {*ε* ∼ N(0,*σ*^2^)}, where *ε* is the vector of independent residuals with a mean of zero and a variance of *σ*^2^ [61], an assumption that is frequently overlooked or not reported in biomedical research. First, nonnormality does not affect the estimation of the parameters if the other assumptions are met but is essential for hypothesis testing (*t*-test and *F*-test) and the construction of the confidence intervals [62]. Therefore, the significance of the parameters and the comparison between products is not reliable if the residual distribution is not normal. On the other hand, the failure of normality may be a sign of model misspecification [63, 64], which in the case of a generic antibiotic suggests that the product dose-response relationship is governed by a different function than the innovator’s, a feature that rules out therapeutic equivalence, because an equivalent generic must display the same pharmacodynamic profile of the innovator. Moreover, the relevance of this apparently “subtle” indicator of nonequivalence was demonstrated by the higher enrichment of *E*. *coli* resistant subpopulation by the generic Farmalogica. Had we overlooked the violation of the normality assumption, none of these data would have been produced, and a radically different conclusion would have been reached.

In summary, we provide here solid experimental evidence confirming the relationship between therapeutic nonequivalence of generic antibiotics and the higher enrichment of bacterial resistance, emphasizing the need for generics with demonstrated instead of assumed *in vivo* equivalence as a key factor to control the problem of resistance worldwide.

## Supporting Information

S1 Fig Sequences of the *bla*_*TEM-1*_ gene and its promoter from *E*. *coli* ATCC 35218, 35218R and the reference sequence. Accession number DQ058146.1.
(DOCX)

S2 Fig Residuals’ plot from the least-squares nonlinear regression of the dose-response relationship of innovator (Wyeth) and generic (Farmalogica) TZP against *E*. *coli* ATCC 35218. (DOCX)

S1 File ARRIVE guidelines checklist. (PDF)

S1 Table Bacterial *in vivo* growth rates estimated by a modified Gompertz’ equation. (DOCX)

S2 Table Percentage of resistance after innovator (Wyeth) and generic (Farmalogica) TZP exposure. The data follow the inverted U shape of the resistance pattern illustrated by panel A of Fig 7.
(DOCX)

S3 Table Percentage of resistance after innovator (Wyeth) and generic (Farmalogica) TZP exposure. The data follow the inverted U shape of the resistance pattern illustrated by panel B of Fig 7.
(DOCX)

S4 Table Percentage of resistance after innovator (Wyeth) and generic (Farmalogica) TZP exposure. The data follow the inverted U shape of the resistance pattern illustrated by panel C of Fig 7.
(DOCX)

S5 Table Percentage of resistance after innovator (Wyeth) and generic (Farmalogica) TZP exposure. The data follow the inverted U shape of the resistance pattern illustrated by panel D of Fig 7.
(DOCX)
