# Supplementary material for: Impact on Bacterial Resistance of Therapeutically Nonequivalent Generics: The Case of Piperacillin-Tazobactam
Source: PLoS One. 2016 May 18;11(5):e0155806. doi: 10.1371/journal.pone.0155806 (PMC4871539; doi:10.1371/journal.pone.0155806)
Supplement: S1 Fig — Accession number DQ058146.1. (DOCX) [file pone.0155806.s001.docx]

**S1 Figure.** Sequences of the *bla_TEM-1_* gene and its promoter from *E. coli* ATCC 35218, 35218R and the reference sequence (Accession number DQ058146.1).

1 10 20 30 40 50 60

Sutcliffe Number ....|....|....|....|....|....|....|....|....|....|....|....|

Reference TEM-1B **TTCTTGAAGACGAAAGGGCCTCGTGATACGCCTATTTTTATAGGTTAATGTCATGATAAT**

35218R ---------------**GGGCCTCG.....................................**

ATCC 35218 -----------------------**.....................................**

70 80 90 100 110 120

....|....|....|....|....|....|....|....|....|....|....|....|

Reference TEM-1B **AATGGTTTCTTAGACGTCAGGTGGCACTTTTCGGGGAAATGTGCGCGGAACCCCTATTTG**

35218R **............................................................**  ATCC 35218 **............................................................**

130 140 150 160 170 180

....|....|....|....|....|....|....|....|....|....|....|....|

Reference TEM-1B **TTTATTTTTCTAAATACATTCAAATATGTATCCGCTCATGAGACAATAACCCTGGTAAAT**

35218R **............................................................**

ATCC 35218 **............................................................**

190 200 210 220 230 240

....|....|....|....|....|....|....|....|....|....|....|....|

Reference TEM-1B **GCTTCAATAATATTGAAAAAGGAAGAGTATGAGTATTCAACATTTTCGTGTCGCCCTTAT**

35218R **............................................................**

ATCC 35218 **............................................................**

Amino acid MetSerIleGlnHisPheArgValAlaLeuIle

Ambler number 3 4 5 6 7 8 9 10 11 12 13

250 260 270 280 290 300

....|....|....|....|....|....|....|....|....|....|....|....|

Reference TEM-1B **TCCCTTTTTTGCGGCATTTTGCCTTCCTGTTTTTGCTCACCCAGAAACGCTGGTGAAAGT**

35218R **............................................................**

ATCC 35218 **............................................................**

Amino acid ProPhePheAlaAlaPheCysLeuProValPheAlaHisProGluThrLeuValLysVal

Ambler number 14 15 16 17 18 19 20 21 22 23 24 25 26 27 28 29 30 31 32 33

310 320 330 340 350 360

....|....|....|....|....|....|....|....|....|....|....|....|

Reference TEM-1B **AAAAGATGCTGAAGATCAGTTGGGTGCACGAGTGGGTTACATCGAACTGGATCTCAACAG**

35218R **............................................................**

ATCC 35218 **............................................................**

Amino acid LysAspAlaGluAspGlnLeuGlyAlaArgValGlyTyrIleGluLeuApsLeuAsnSer

Ambler Number 34 35 36 37 38 39 40 41 42 43 44 45 46 47 48 49 50 51 52 53

370 380 390 400 410 420

....|....|....|....|....|....|....|....|....|....|....|....|

Reference TEM-1B **CGGTAAGATCCTTGAGAGTTTTCGCCCCGAAGAACGTTTTCCAATGATGAGCACTTTTAA**

35218R **............................................................**

ATCC 35218 **............................................................**

Amino acid GlyLysIleLeuGluSerPheArgProGluGluArgPheProMetMetSerThrPheLys

Ambler number 54 55 56 57 58 59 60 61 62 63 64 65 66 67 68 69 70 71 72 73

430 440 450 460 470 480

....|....|....|....|....|....|....|....|....|....|....|....|

Reference TEM-1B **AGTTCTGCTATGTGGTGCGGTATTATCCCGTGTTGACGCCGGGCAAGAGCAACTCGGTCG**

35218R **............................................................**

ATCC 35218 **............................................................**

Amino Acid ValLeuLeuCysGlyAlaValLeuSerArgValAspAlaGlyGlnGluGlnLeuGlyArg

Ambler number 74 75 76 77 78 79 80 81 82 83 84 85 86 87 88 89 90 91 92 93

490 500 510 520 530 540

Sutcliffe Number ....|....|....|....|....|....|....|....|....|....|....|....|

Reference TEM-1B **CCGCATACACTATTCTCAGAATGACTTGGTTGAGTACTCACCAGTCACAGAAAAGCATCT**

35218R **............................................................**

ATCC 35218 **............................................................**

Amino Acid ArgIleHisTyrSerGlnAsnAspLeuValGluTyrSerProValThrGluLysHisLeu

Ambler Number 94 95 96 97 98 99 100 102 104 106 108 110 112

550 560 570 580 590 600

....|....|....|....|....|....|....|....|....|....|....|....|

Reference TEM-1B **TACGGATGGCATGACAGTAAGAGAATTATGCAGTGCTGCCATAACCATGAGTGATAACAC**

35218R **............................................................**

ATCC 35218 **............................................................**

Amino Acid ThrAspGlyMetThrValArgGluLeuCysSerAlaAlaIleThrMetSerAspAsnThr

Ambler Number 114 116 118 120 122 124 126 128 130 132

610 620 630 640 650 660

....|....|....|....|....|....|....|....|....|....|....|....|

Reference TEM-1B **TGCTGCCAACTTACTTCTGACAACGATCGGAGGACCGAAGGAGCTAACCGCTTTTTTGCA**

35218R **............................................................**

ATCC 35218 **............................................................**

Amino Acid AlaAlaAnsLeuLeuLeuThrThrIleGlyGlyProLysGluLeuThrAlaPheLeuHis

Ambler Number 134 136 138 140 142 144 146 148 150 152

670 680 690 700 710 720

....|....|....|....|....|....|....|....|....|....|....|....|

Reference TEM-1B **CAACATGGGGGATCATGTAACTCGCCTTGATCGTTGGGAACCGGAGCTGAATGAAGCCAT**

35218R **............................................................**

ATCC 35218 **............................................................**

Amino Acid AsnMetGlyAspHisValThrArgLeuAspArgTrpGluProGluLeuAsnGluAlaIle

Ambler Number 154 156 158 160 162 164 166 168 170 172

730 740 750 760 770 780

....|....|....|....|....|....|....|....|....|....|....|....|

Reference TEM-1B **ACCAAACGACGAGCGTGACACCACGATGCCTGCAGCAATGGCAACAACGTTGCGCAAACT**

35218R **............................................................**

ATCC 35218 **............................................................**

Amino Acid ProAsnAspGluArgAspThrThrMetProAlaAlaMetAlaThrThrLeuArgLysLeu

Ambler Number 174 176 178 180 182 184 186 188 190 192

790 800 810 820 830 840

....|....|....|....|....|....|....|....|....|....|....|....|

Reference TEM-1B **ATTAACTGGCGAACTACTTACTCTAGCTTCCCGGCAACAATTAATAGACTGGATGGAGGC**

35218R **............................................................**

ATCC 35218 **............................................................**

Amino Acid LeuThrGlyGluLeuLeuThrLeuAlaSerArgGlnGlnLeuIleAspTrpMetGluAla

Ambler Number 194 196 198 200 202 204 206 208 210 212

850 860 870 880 890 900

....|....|....|....|....|....|....|....|....|....|....|....|

Reference TEM-1B **GGATAAAGTTGCAGGACCACTTCTGCGCTCGGCCCTTCCGGCTGGCTGGTTTATTGCTGA**

35218R **............................................................**

ATCC 35218 **............................................................**

Amino Acid AspLysValAlaGlyProLeuLeuArgSerAlaLeuProAlaGlyTrpPheIleAlaAsp

Ambler Number 214 216 218 220 222 224 226 228 230 232

910 920 930 940 950 960

....|....|....|....|....|....|....|....|....|....|....|....|

Reference TEM-1B **TAAATCTGGAGCCGGTGAGCGTGGGTCTCGCGGTATCATTGCAGCACTGGGGCCAGATGG**

35218R **............................................................**

ATCC 35218 **............................................................**

Amino Acid LysSerGlyAlaGlyGluArgGlySerArgGlyIleIleAlaAlaLeuGlyProAspGly

Ambler Number 234 236 238 241 243 245 247 249 251 254

Sutcliffe Number 970 980 990 1000 1010 1020

....|....|....|....|....|....|....|....|....|....|....|....|

Reference TEM-1B **TAAGCCCTCCCGTATCGTAGTTATCTACACGACGGGGAGTCAGGCAACTATGGATGAACG**

35218R **............................................................**

ATCC 35218 **............................................................**

Amino Acid LysProSerArgIleValValIleTyrThrThrGlySerGlnAlaThrMetAspGluArg

Ambler Number 256 258 260 262 264 266 268 270 272 274

1030 1040 1050 1060 1070

Sutcliffe Number ....|....|....|....|....|....|....|....|....|....|

Reference TEM-1B **AAATAGACAGATCGCTGAGATAGGTGCCTCACTGATTAAGCATTGGTAA**

35218R ............................---------------------

ATCC 35218 .....................................------------

Amino Acid AsnArgGlnIleAlaGluIleGlyAlaSerLeuIleLysHisTrp

Ambler Number 276 278 280 282 284 286 288 290

The promoter spans from Sutcliffe bases 1 to 208 (blue letters). Bases C32, T147, G162 and G175, corresponding to the *P3* promoter are underlined (green), while the -35 (TTCAAA) and -10 (GACAAT) regions are blue and underlined. The coding region (black letters) goes from bases 209 to 1066, encoding 286 amino acids (Ambler 3 to 290, there are no Ambler 1, 2, 239 and 253 positions in TEM-1). The stop codon (TAA) is at the end in red.
