# Supplementary material for: Impact on Bacterial Resistance of Therapeutically Nonequivalent Generics: The Case of Piperacillin-Tazobactam
Source: PLoS One. 2016 May 18;11(5):e0155806. doi: 10.1371/journal.pone.0155806 (PMC4871539; doi:10.1371/journal.pone.0155806)
Supplement: S2 Fig — (DOCX) [file pone.0155806.s002.docx]

**S2 Fig.** Residuals’ plot from the least-squares nonlinear regression of the dose-response relationship of innovator (Wyeth) and generic (Farmalogica) TZP against *E. coli* ATCC 35218.
