# Supplementary material for: Impact on Bacterial Resistance of Therapeutically Nonequivalent Generics: The Case of Piperacillin-Tazobactam
Source: PLoS One. 2016 May 18;11(5):e0155806. doi: 10.1371/journal.pone.0155806 (PMC4871539; doi:10.1371/journal.pone.0155806)
Supplement: S1 Table — (DOCX) [file pone.0155806.s004.docx]

**S1 Table**. Bacterial *in vivo* growth rates estimated by a modified Gompertz’ equation.

| Strain | Inoculum (log_10_ CFU/mL) | Mean (SEM) growth rate in vivo per hour | Mean (SEM) total growth in vivo in log_10_ CFU/g |
| --- | --- | --- | --- |
| *E. coli* ATCC 35218 pure | 5 (pure) | 0.64 (0.16) | 5.29 (0.16) |
| *E. coli* 35218R pure | 5 (pure) | 0.62 (0.08) | 4.39 (0.08) |
| *E. coli* ATCC 35218 mixed | 7 (mixed) | 0.56 (0.13) | 3.60 (0.13) |
| *E. coli* 35218R mixed | 5 (mixed) | 0.21 (0.15) | 2.12 (0.15) |
| *E. coli* 35218Δ*bla* pure | 5 (pure) | 0.75 (0.1) | 5.54 (0.10) |
| *S. aureus* GRP-0057 | 5 (pure) | 0.43 (0.1) | 3.86 (0.10) |
| *E. faecium* ATCC 51559 | 5 (pure) | 0.66 (0.04) | 4.04 (0.07) |
| *P. aeruginosa* GPR-0019 | 5 (pure) | 0.50 (0.07) | 5.00 (0.07) |
| *E. coli* SIG-1 | 6 (pure) | 0.73 (0.05) | 3.62 (0.10) |
