# Supplementary material for: Impact on Bacterial Resistance of Therapeutically Nonequivalent Generics: The Case of Piperacillin-Tazobactam
Source: PLoS One. 2016 May 18;11(5):e0155806. doi: 10.1371/journal.pone.0155806 (PMC4871539; doi:10.1371/journal.pone.0155806)
Supplement: S2 Table — The data follow the inverted U shape of the resistance pattern illustrated by panel A of Fig 7. (DOCX) [file pone.0155806.s005.docx]

**S2 Table**. Percentage of resistance after innovator (Wyeth) and generic (Farmalogica) TZP exposure. The data follow the inverted U shape of the resistance pattern illustrated by panel A of Fig 7.

| Piperacillin  24h Dose (mg/kg) | Wyeth  % resistance  *w*Mean (*w*SD) | Farmalogica  % resistance  *w*Mean (*w*SD) | P value* |
| --- | --- | --- | --- |
| 5120 | 3.59 (1.49) | 2.25 (2.13) | 0.8777 |
| 2560 | 2.30 (0.65) | 2.69 (2.41) | 0.9643 |
| 1280 | 3.65 (1.72) | 3.07 (1.18) | 0.9469 |
| 640 | 12.86 (16.98) | 92.21 (13.01) | **<0.0001** |
| 320 | 15.08 (23.10) | 18.67 (19.16) | 0.6452 |
| 160 | 9.68 (10.29) | 18.87 (18.81) | 0.2941 |
| 80 | 2.65 (2.29) | 2.31 (2.44) | 0.9688 |

*Student’s t test followed by Holm-Sidak post-hoc multiple comparisons test.
