# Supplementary material for: Impact on Bacterial Resistance of Therapeutically Nonequivalent Generics: The Case of Piperacillin-Tazobactam
Source: PLoS One. 2016 May 18;11(5):e0155806. doi: 10.1371/journal.pone.0155806 (PMC4871539; doi:10.1371/journal.pone.0155806)
Supplement: S3 Table — The data follow the inverted U shape of the resistance pattern illustrated by panel B of Fig 7. (DOCX) [file pone.0155806.s006.docx]

**S3 Table.** Percentage of resistance after innovator (Wyeth) and generic (Farmalogica) TZP exposure. The data follow the inverted U shape of the resistance pattern illustrated by panel B of Fig 7.

| Piperacillin  24h Dose (mg/kg) | Wyeth  % resistance  *w*Mean (*w*SD) | Farmalogica  % resistance  *w*Mean (*w*SD) | P value* |
| --- | --- | --- | --- |
| 5120 | 0.82 (0.03) | 1.22 (1.87) | 0.8470 |
| 2560 | 0.48 (0.92) | 1.55 (0.88) | 0.6064 |
| 1280 | 1.57 (1.26) | 1.60 (0.60) | 0.9884 |
| 640 | 0.52 (0.22) | 10.52 (6.93) | **<0.0001** |
| 320 | 5.32 (3.28) | 3.81 (0.40) | 0.4683 |
| 160 | 3.01 (0.99) | 3.85 (0.74) | 0.6856 |
| 80 | 4.24 (4.31) | 1.57 (1.55) | 0.2042 |

*Student’s t test followed by Holm-Sidak post-hoc multiple comparisons test.
