# Supplementary material for: Impact on Bacterial Resistance of Therapeutically Nonequivalent Generics: The Case of Piperacillin-Tazobactam
Source: PLoS One. 2016 May 18;11(5):e0155806. doi: 10.1371/journal.pone.0155806 (PMC4871539; doi:10.1371/journal.pone.0155806)
Supplement: S4 Table — The data follow the inverted U shape of the resistance pattern illustrated by panel C of Fig 7. (DOCX) [file pone.0155806.s007.docx]

**S4 Table**. Percentage of resistance after innovator (Wyeth) and generic (Farmalogica) TZP exposure. The data follow the inverted U shape of the resistance pattern illustrated by panel C of Fig 7.

| Piperacillin  24h Dose (mg/kg) | Wyeth  % resistance  *w*Mean (*w*SD) | Farmalogica  % resistance  *w*Mean (*w*SD) | P value* |
| --- | --- | --- | --- |
| 5120 | 0.35 (0.77) | 0.29 (0.83) | 0.9317 |
| 2560 | 0.57 (0.05) | 0.84 (0.54) | 0.7099 |
| 1280 | 0.67 (0.59) | 4.50 (2.30) | **<0.0001** |
| 640 | 1.30 (0.31) | 1.98 (0.34) | 0.3441 |
| 320 | 1.71 (0.37) | 1.37 (0.28) | 0.6387 |
| 160 | 1.32 (0.83) | 1.09 (0.22) | 0.7991 |
| 80 | 0.44 (0.28) | 0.99 (1.47) | 0.4432 |

*Student’s t test followed by Holm-Sidak post-hoc multiple comparisons test.
