# Supplementary material for: Impact on Bacterial Resistance of Therapeutically Nonequivalent Generics: The Case of Piperacillin-Tazobactam
Source: PLoS One. 2016 May 18;11(5):e0155806. doi: 10.1371/journal.pone.0155806 (PMC4871539; doi:10.1371/journal.pone.0155806)
Supplement: S5 Table — The data follow the inverted U shape of the resistance pattern illustrated by panel D of Fig 7. (DOCX) [file pone.0155806.s008.docx]

**S5 Table**. Percentage of resistance after innovator (Wyeth) and generic (Farmalogica) TZP exposure. The data follow the inverted U shape of the resistance pattern illustrated by panel D of Fig 7.

| Piperacillin  24h Dose (mg/kg) | Wyeth  % resistance  *w*Mean (*w*SD) | Farmalogica  % resistance  *w*Mean (*w*SD) | Procaps  % resistance  *w*Mean (*w*SD) | P value  Wyeth  Farmalogica* | P value  Wyeth  Procaps* |
| --- | --- | --- | --- | --- | --- |
| 2560 | 0.23 (0.08) | 0.34 (0.24) | 0.32 (0.19) | 0.3992 | 0.4389 |
| 1280 | 0.06 (0.16) | 0.15 (0.06) | 0.22 (0.20) | 0.4881 | 0.1801 |
| 640 | 0.48 (0.13) | 0.27 (0.04) | 0.17 (0.03) | 0.1210 | 0.0173 |
| 320 | 0.33 (0.01) | 0.81 (0.10) | 0.17 (0.07) | **0.0025** | 0.1801 |
| 160 | 0.14 (0.03) | 0.46 (0.21) | 0.35 (0.01) | 0.0258 | 0.0863 |
| 80 | 0.25 (0.14) | 0.18 (0.06) | 0.09 (0.01) | 0.5882 | 0.1801 |

*Student’s t test followed by Holm-Sidak post-hoc multiple comparisons test. The only significant difference is between Wyeth and Farmalogica at 320 mg/kg (bold). The P value between Wyeth and Procaps at 640 mg/kg is not significant after the multiple comparison test (alpha error <0.01).
